# Retraction: Sequence rules for gold-binding peptides

**DOI:** 10.1039/d6ra90162j

**Published:** 2026-10-28

**Authors:** Jose Isagani B. Janairo

**Affiliations:** a Department of Biology, De La Salle University 2401 Taft Avenue Manila 0922 Philippines jose.isagani.janairo@dlsu.edu.ph

## Abstract

Retraction of ‘Sequence rules for gold-binding peptides’ by Jose Isagani B. Janairo *et al.*, *RSC Adv.*, 2023, **13**, 21146–21152, https://doi.org/10.1039/d3ra04269c.

I, the author, hereby wholly retract this *RSC Advances* article due to concerns that the conclusions are fundamentally undermined by errors in the training data.

Upon investigation, the author has discovered that many sequences for the class A peptides used in the dataset have been found to be duplicates. Because of this, the findings and conclusion of the study are no longer valid as the machine learning model was trained on incorrect data.

In this study, the author used 860 peptides, which were divided into two classes, Class A and Class B, each with 430 sequences. The dataset used was deposited in github. Upon further investigation, for the Class A peptides, it has been discovered that instead of 430 unique sequences, the dataset contained only 38 unique sequences for Class A, with these sequences being repeated several times.

Based on the high degree of sequence repetition, the results of the machine learning output are incorrect. Given the significance of these concerns, the conclusions presented in this paper are no longer reliable.

An independent expert has reviewed this case and has deemed that retraction is the most appropriate course of action.

The Royal Society of Chemistry apologises for these errors and any consequent inconvenience to authors and readers.

Signed: Jose Isagani B. Janairo

Date: 21st October 2026

Retraction endorsed by Laura Fisher, Executive Editor, *RSC Advances*

